# The Efficacy and Safety of a Combination of Thiocolchicoside and Etoricoxib in Low Back Pain (ESCoTEL): A Randomized Active-Controlled Trial

**DOI:** 10.7759/cureus.47621

**Published:** 2023-10-25

**Authors:** Arnab Karmakar, Sumit Arora, Rajat Singal, Sandip Mitra, Gitika, Manipa Saha, Monjori Mitra

**Affiliations:** 1 Orthopaedics, Institute of Post-Graduate Medical Education and Research and Seth Sukhlal Karnani Memorial Hospital, Kolkata, IND; 2 Orthopaedics, Maulana Azad Medical College, New Delhi, IND; 3 Medical Affairs, Mankind Pharma Ltd., New Delhi, IND; 4 Medical Affairs, Medclin Research Pvt. Ltd., Kolkata, IND; 5 Pediatrics, Institute of Child Health, Kolkata, IND

**Keywords:** fixed-dose combination (fdc), thiocolchicoside, randomized controlled trial (rct), low back pain (lbp), etoricoxib

## Abstract

Background

Low back pain (LBP) is a global health concern. Management of LBP aims at pain relief facilitating improvement of functional ability. Non-steroidal anti-inflammatory drugs (NSAIDs) are the first line of therapy. However, the selection of NSAIDs is challenging given the range of underlying etiologies and severity. The current study aimed to compare the efficacy and safety of two available fixed-dose combinations (FDCs), namely, a dual FDC (DFC) of etoricoxib (60 mg) and thiocolchicoside (4 mg) versus a triple FDC (TFC) of chlorzoxazone (500 mg), diclofenac (50 mg), and paracetamol (325 mg).

Methodology

A total of 200 eligible adult subjects aged 18-70 years with a history of LBP and muscle spasm for ≤14 days and Wong-Baker Faces Pain score >4 were enrolled after obtaining written informed consent and randomized in a 1:1 allocation ratio to be treated with either DFC or TFC for 28 days. Efficacy was assessed based on the change in score from baseline (before treatment) to day 28 on the Wong-Baker Faces Pain Scale and the Oswestry Disability Index (ODI) questionnaire, as well as the proportion of subjects who improved upon treatment. Safety was assessed based on adverse events and clinical laboratory test results.

Results

A significant decrease in pain intensity (p < 0.001) and significant improvement in functional ability (p < 0.001) was observed after treatment with either DFC or TFC. The decrease in Wong-Baker Faces Pain score and ODI, from baseline, was comparable between the treatment groups. However, more subjects with very severe pain at baseline showed ≥30% improvement upon treatment with DFC than with TFC (~25% versus ~12%; p = 0.172). Also, significantly more crippled subjects with very severe functional disability showed improvement in the DFC group compared to the TFC group (~26% versus ~4%; p = 0.008). No adverse events or clinically relevant laboratory test results were evident.

Conclusions

Both DFC and TFC were comparable in efficacy and safety for the management of recent-onset LBP. However, significantly more subjects with very severe pain or functional disability showed improvement after 28 days when treated with DFC compared to TFC.

## Introduction

Low back pain (LBP) is a spectrum of pain disorders that might be overlapping, often with distinct sources [1,2]. Globally, it is the leading cause of years lived with disability (YLDs). Based on the Global Burden of Disease, Injuries, and Risk Factors Study (GBD) 2019, musculoskeletal disorders were the most prevalent with 1.71 billion affected people, with LBP ranking the highest in 134 of 204 countries [2]. The estimates of incidence, prevalence, and disability-adjusted life years (DALYs) of LBP, as of 2020, were 245.9 million cases/year (3.2%), 577.0 million cases (7.6%), and 64.9 million DALYs (2.6%), respectively, all of which increased by ~50% during the last 20 years [3]. The pooled annual and lifetime prevalence of LBP in India was reported as 51% and 66%, respectively, in a recent systematic review [4].

A large proportion of LBP cases resolve without formal diagnosis; however, diagnosis is recommended based on physical examination, recording of history, response of patients to questionnaires, and medical imaging. Prevention strategies are not widely in practice primarily due to a lack of acknowledgment of non-anatomical factors in the onset and progress of LBP. Therefore, management strategies are of paramount importance [1]. The aim of LBP management is to alleviate pain quickly and improve functional ability. Non-steroidal anti-inflammatory drugs (NSAIDs) are the first line of treatment. However, the challenge lies in deciding which NSAIDs to administer for greater and faster symptomatic relief.

This study was designed to investigate the cumulative effects of the administration of NSAIDs and muscle relaxants in patients with LBP. The test medication of this study is a fixed-dose combination (FDC) of the NSAID etoricoxib (60 mg) and the muscle relaxant thiocolchicoside (4 mg). Etoricoxib is a selective cyclooxygenase-2 inhibitor that also confers gastroprotection. Thiocolchicoside acts on inhibitory neurotransmitters; it has anti-inflammatory and analgesic effects and does not cause sedation [5]. The use of NSAIDs and/or muscle relaxants is recommended by international guidelines [6-10]. In an overview of clinical practice guidelines published between 2008 and 2017, Oliveira et al. noted that 93% of guidelines recommended the use of NSAIDs in LBP considering the risk of adverse events and 54% recommended the use of muscle relaxants [11].

At the initiation of this study, there were no reports, to our knowledge, of an FDC of etoricoxib and thiocolchicoside. However, a recent study published from India reported faster relief of recent-onset painful muscle spasms upon treatment with an FDC of etoricoxib and thiocolchicoside compared to thiocolchicoside monotherapy in adult patients [5]. In our study, we compared this dual FDC (DFC) of etoricoxib and thiocolchicoside to a triple FDC (TFC) of chlorzoxazone (muscle relaxant; 500 mg), diclofenac (NSAID; 50 mg), and paracetamol (analgesic; 325 mg). The two FDCs were compared for efficacy and safety.

## Materials and methods

This open-label, randomized controlled trial (RCT) was conducted from July 2022 to January 2023 at two sites in India, namely, Maulana Azad Medical College (New Delhi) and Institute of Post-Graduate Medical Education and Research and Seth Sukhlal Karnani Memorial Hospital (Kolkata). The study was conducted in accordance with the principles of the Declaration of Helsinki and Good Clinical Practice guidelines for clinical trials on Pharmaceutical Products in India, as mentioned in the New Drugs and Clinical Trials Rules 2019 (issued by the Central Drugs Standard Control Organization, Ministry of Health, Government of India), as well as in compliance with the requirements of the clinical study protocol approved by the Ethics Committees of the study sites. Written informed consent was obtained from all subjects before enrollment. The trial was registered with the Clinical Trials Registry of India on July 4, 2022 (reference number: CTRI/2022/07/043690).

To detect a difference of 13% in pain severity on the Wong-Bakers Faces Pain scale between the two treatment groups, assuming a power of 80%, a level of significance of 5%, and considering a dropout rate of 5%, 100 subjects were required in each treatment group and a total population of 200 subjects was required in the study. Eligible subjects were of either sex, aged 18-70 years (both inclusive), having a history of LBP and muscle spasm for ≤14 days, and with a Wong-Baker Faces Pain score >4 at baseline. Subjects with back pain due to fractures, malignancy, infection, abnormal metabolism, osteoarthritis of the hip or any other disease, or with back pain originating or referred from other organs were excluded from the study. Subjects with asthma or other allergic disorders, those with a history of peptic ulceration or gastrointestinal bleeding or severe dyspepsia, or those with a known allergy to NSAIDs/skeletal muscle relaxants or any component of the study product were also excluded. Eligible subjects were randomized using permuted block randomization by 1:1 allocation to be treated with either DFC (etoricoxib (60 mg) + thiocolchicoside (4 mg)) or TFC (chlorzoxazone (500 mg) + diclofenac (50 mg) + paracetamol (325 mg)). Both medications were consumed orally once daily in the morning for 28 days.

Efficacy was assessed based on the Wong-Baker Faces Pain Scale [12], which enables patients to choose from a visual representation of pain gradation, and the Oswestry Disability Index (ODI) questionnaire [13], which is considered the gold standard for assessment of the degree of disability in recent-onset LBP. The primary endpoints of the study were changes in score on both of these from baseline to day 28. The secondary endpoints were the corresponding changes from baseline to day 14, as well as the proportion of subjects showing improvement on day 28 (referred to as responders). Assessment of safety was done based on adverse events and clinical laboratory test results (hemogram, liver and kidney function tests, electrolyte levels).

All statistical methods were based on the International Conference on Harmonization E9 document Statistical Principles for Clinical Trials and all statistical analyses were done using SPSS version 28.0.1.1 (IBM Corp., Armonk, NY, USA). All hypothesis testing was performed at the 5% significance level. The Central Limit Theorem was assumed for data normality. This theorem states that the distribution of sample means approximates a normal distribution as the sample size gets larger, regardless of the population’s distribution. Sample sizes ≥30 are often considered sufficient for the theorem to hold true. An independent t-test was used to compare demographic variables between the two groups. Repeated-measures analysis of variance and post-hoc pairwise (with Bonferroni correction) was used to analyze scores on the Wong-Baker Faces Pain scale and ODI. Categorical data between treatment groups was analyzed using Z-test.

## Results

**Figure 1:**
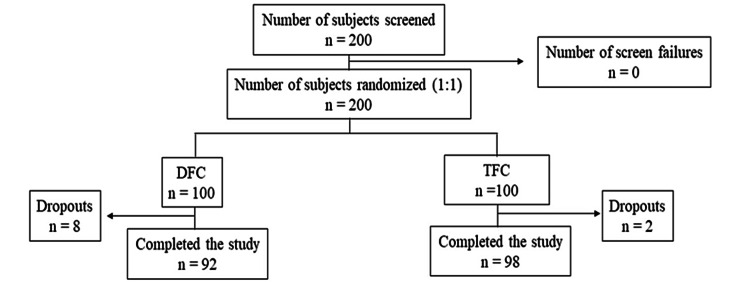
Subject disposition. DFC = dual fixed combination; TFC = triple fixed combination

A total of 200 subjects were screened who met the eligibility criteria and were randomized. In total, 100 subjects were enrolled in each of the two treatment groups (Figure 1).

The proportion of female subjects in DFC and TFC was 55/100 (55%) and 56/100 (56%), respectively. Most subjects (33%) were 41-50 years old. The demographic characteristics of subjects in both groups were comparable at baseline, with no statistically significant differences between the groups (Table 1).

**Table 1:** Baseline demographic characteristics of enrolled subjects. DFC = dual fixed combination; SD = standard deviation; SEM = standard error of the mean; TFC = triple fixed combination

Parameter	Measure	DFC (N = 100)	TFC (N = 100)	P-value
Age (years)	Mean ± SD	41.67 ± 11.78	41.36 ± 10.78	0.424
	SEM	1.178	1.078	
	Median (minimum, maximum)	41.84 (18.58, 69.67)	42.54 (18.83, 66.67)	
Height (in cm)	Mean ± SD	160.16 ± 10.00	160.24 ± 8.36	0.475
	SEM	1	0.836	
	Median (minimum, maximum)	161 (124, 182)	158.85 (141, 183)	
Weight (in kg)	Mean ± SD	61.60 ± 8.20	63.02 ± 9.43	0.128
	SEM	0.820	0.943	
	Median (minimum, maximum)	61 (41, 80)	63 (44, 90)	
Gender	Male n (%)	45 (45%)	44 (44%)	1
	Female n (%)	55 (55%)	56 (56%)	

**Figure 2:**
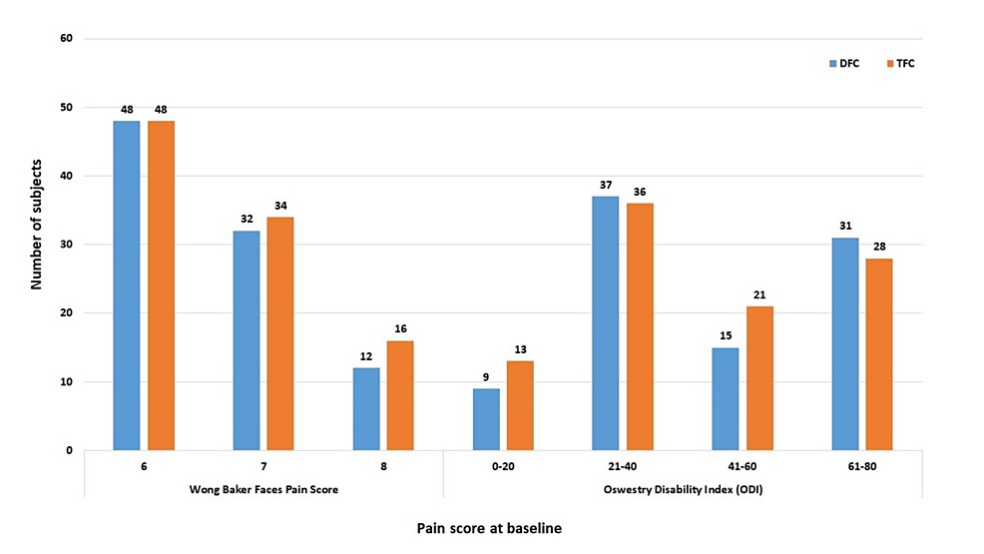
Distribution of pain score and Oswestry Disability Index at baseline in subjects who completed the study. DFC = dual fixed combination; ODI = Oswestry Disability Index; TFC = triple fixed combination

A total of 10 subjects were lost to follow-up; 190 subjects (DFC, 92; TFC, 98) completed all visits and were considered for efficacy analysis (Figure 1). When classified based on the Wong-Baker Faces Pain score, most subjects had a score of 6 at baseline corresponding to moderate-to-severe pain (DFC, 48/92 (52.17%); TFC, 48/98 (48.98%)). Some subjects reported very severe pain, that is, a score of 7-8 (DFC, 44/92 (47.83%); TFC, 50/98 (51.02%)). Based on the ODI scale, most subjects had moderate disability, that is a score in the range of 21-40 (DFC, 37/92 (40.22%); TFC, 36/98 (36.73%)). There were some subjects who were crippled, that is, LBP had an impact on all aspects of their daily living and work; these subjects had an ODI in the range of 61-80 (DFC, 31/92 (33.70%); TFC, 28/98 (28.57%)). The distribution of the scores was comparable between subjects from both treatment groups (Figure 2).

After 28 days of treatment, subjects from both treatment groups showed a significant decrease in pain intensity (based on the decrease in Wong-Baker Faces Pain score; p < 0.001) and a significant improvement in functional ability (based on a decrease in ODI; p < 0.001). The same was true for early improvement in pain intensity and functional ability on day 14 (p < 0.001 for both groups) (Tables 2, 3).

**Table 2:** Change in Wong-Baker Faces Pain score. ***: p<0.001. CI = confidence interval; DFC = dual fixed combination; SD = standard deviation; SE = standard error; SEM = standard error of the mean; TFC = triple fixed combination

	DFC (N = 92)		TFC (N = 98)	
	Baseline	Day 14	Baseline	Day 14
Mean ± SD	6.60 ± 0.71	5.34 ± 1.14	6.67 ± 0.74	5.19 ± 1.45
SEM	0.074	0.119	0.075	0.146
Median (minimum, maximum)	6 (6, 8)	5.50 (2, 8)	7 (6, 8)	5 (0, 8)
Mean change± SE (95% CI)	1.272 ± 0.104 (1.021, 1.523)		1.480 ± 0.101 (1.236, 1.723)	
Percentage decrease from baseline	19.27%		22.21%	
P-value	<0.001***		<0.001***	
	Baseline	Day 28	Baseline	Day 28
Mean ± SD	6.60 ± 0.71	4.50 ± 1.494	6.67 ± 0.74	4.60 ± 1.55
SEM	0.074	0.156	0.075	0.157
Median (minimum, maximum)	6 (6, 8)	5 (1, 8)	7 (6, 8)	5 (0, 8)
Mean change± SE (95% CI)	2.109 ± 0.132 (1.791, 2.427)		2.071 ± 0.127 (1.763, 2.379)	
Percentage decrease from baseline	31.95%		31.05%	
P-value	<0.001***		<0.001***	

**Table 3:** Change in Oswestry Disability Index. ***: p < 0.001. CI = confidence interval; DFC = dual fixed combination; SD = standard deviation; SE = standard error; SEM = standard error of the mean; TFC = triple fixed combination

	DFC (N = 92)		TFC (N = 98)	
	Baseline	Day 14	Baseline	Day 14
Mean ± SD	44.75 ± 20.70	38.22 ± 22.09	44.31 ± 21.58	37.82 ± 23.70
SEM	2.158	2.303	2.180	2.394
Median (minimum, maximum)	39 (16, 77.78)	37 (8, 77.78)	40.22 (6, 80)	35.89 (2, 80)
Mean change± SE (95% CI)	6.524 ± 0.707 (4.817, 8.232)		6.485 ± 0.685 (4.831, 8.139)	
Percentage decrease from baseline	14.58%		14.63%	
P-value	<0.001***		<0.001***	
	Baseline	Day 28	Baseline	Day 28
Mean ± SD	44.75 ± 20.70	33.63 ± 22.27	44.31 ± 21.58	33.41 ± 24.90
SEM	2.158	2.321	2.180	2.515
Median (minimum, maximum)	39 (16, 77.78)	34.78 (4, 77.78)	40.22 (6, 80)	28 (0, 77.78)
Mean change± SE (95% CI)	11.118 ± 0.915 (8.907, 13.329)		10.902 ± 0.887 (8.760, 13.044)	
Percentage decrease from baseline	24.84%		24.60%	
P-value	<0.001***		<0.001***	

The overall percentages of decrease in Wong-Baker Faces Pain score (DFC, ~32%; TFC, ~31%) and ODI (~25% in both groups) were comparable between the groups. There were a total of 174 responders (DFC, 85/92 (~92%); TFC, 89/98 (~91%)) with respect to Wong-Baker Faces Pain score and 162 responders with respect to ODI (DFC 79/92 (~86%); TFC, 83/98 (~85%)). The proportion of responders in each component of the ODI questionnaire is presented in Table 4. The response in both treatment groups was comparable.

**Table 4:** Proportion of responders in each component of the Oswestry Disability Index questionnaire. DFC = dual fixed combination; TFC = triple fixed combination Note: Proportions are presented as n/N (%), where n is the number of responders among the total N number of subjects who responded to the question at baseline.

Question number	Assessment of functional ability	DFC	TFC
1	Pain intensity	72/92 (78.26%)	78/97 (80.41%)
2	Personal care	35/91 (38.46%)	36/93 (38.71%)
3	Lifting	45/91 (49.45%)	48/98 (48.98%)
4	Walking	42/92 (45.65%)	40/96 (41.67%)
5	Sitting	46/90 (51.11%)	49/95 (51.58%)
6	Standing	53/89 (59.55%)	57/95 (60%)
7	Sleeping	40/80 (50%)	49/87 (56.32%)
8	Sex life	8/20 (40%)	10/20 (50%)
9	Social life	26/69 (37.68%)	21/73 (28.77%)
10	Traveling	44/91 (48.35%)	44/93 (47.31%)

**Figure 3:**
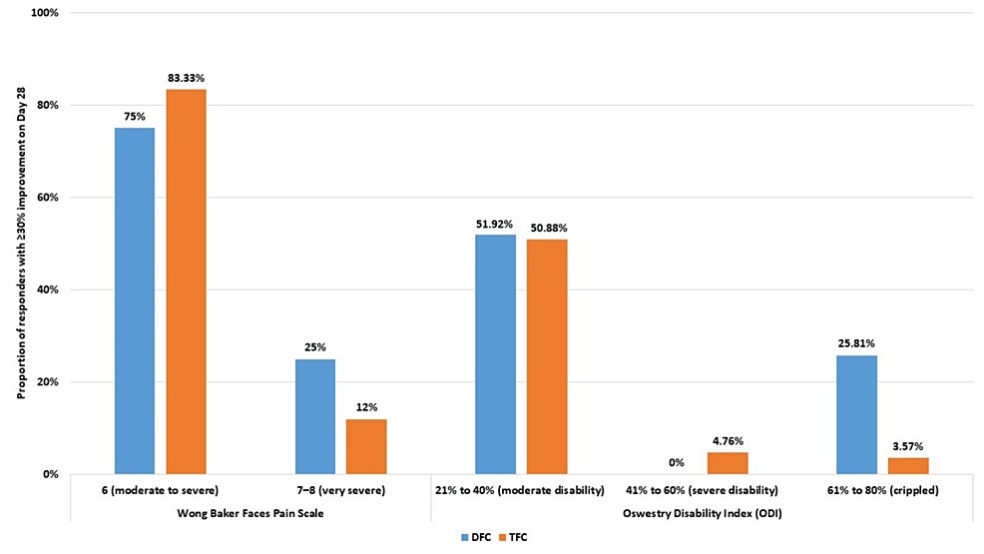
Percentage of subjects with improvement after 28 days of treatment. DFC = dual fixed combination; ODI = Oswestry Disability Index; TFC = triple fixed combination

We analyzed the proportion of responders with ≥30% improvement among subjects classified based on the severity of pain and functional disability. For subjects with pain of moderate-to-severe intensity at baseline (Wong-Baker Faces Pain score of 6), the proportion of responders with ≥30% improvement was more in the TFC group compared to the DFC group, although the difference was not statistically significant (~83% versus ~75%; p = 0.156). However, a higher proportion of subjects with very severe pain (baseline Wong-Baker Faces Pain score of 7-8) in the DFC group showed ≥30% improvement compared to the TFC group (~25% versus ~12%, p=0.172). Among subjects with moderate disability (baseline ODI of 21-40), the proportion of responders with ≥30% improvement was comparable between the treatment groups, while only one subject with severe disability (baseline ODI of 41-60) showed ≥30% improvement. However, among subjects who were classified as crippled (baseline ODI of 61 -80), the proportion of responders with ≥30% improvement was significantly more in the DFC group compared to that in the TFC group (~26% versus ~4%, p = 0.008). Thus, the DFC proved to be beneficial to more subjects with very severe pain compared to the TFC (Table 5 and Figure 3).

**Table 5:** Proportion of subjects with improvement after 28 days of treatment. *: p < 0.05; **: p < 0.01. CI = confidence interval; DFC = dual fixed combination; TFC = triple fixed combination Note: Proportions are presented as n/N (%), where n is the number of responders or responders with ≥30% improvement (as mentioned) among the total N number of subjects with the mentioned score(s) at baseline.

Scale	Score at baseline		DFC	TFC	Z-statistic	Percentage of difference	(95% CI), P-value
Wong-Baker Faces Pain Scale	6 (moderate to severe)	Responders, n/N (%)	44/48 (91.67%)	44/48 (91.67%)	0	0%	(-0.110, 0.110) , 0.999
		Responders with ≥30% improvement, n/N (%)	36/48 (75%)	40/48 (83.33%)	-1.005	-8.33%	(-0.265, 0.099), 0.156
	7–8 (very severe)	Responders, n/N (%)	41/44 (93.18%)	45/50 (90.00%)	0.551	3.18%	(-0.101, 0.164), 0.291
		Responders with ≥30% improvement, n/N (%)	11/44 (25%)	6/50 (12%)	1.365	13%	(-0.047, 0.307), 0.172
Oswestry Disability Index	21% to 40% (moderate disability)	Responders, n/N (%)	41/52 (78.85%)	49/57 (85.96%)	-0.978	-7.11%	(-0.232, 0.090), 0.163
		Responders with ≥30% improvement, n/N (%)	27/52 (51.92%)	29/57 (50.88%)	0.109	1.04%	(-0.187, 0.208), 0.456
	41% to 60% (severe disability)	Responders, n/N (%)	7/15 (46.67%)	14/21 (66.67%)	-1.2	-20%	( -0.580, 0.180), 0.115
		Responders with ≥30% improvement, n/N (%)	0/15 (0%)	1/21 (4.76%)	-0.857	-4.76%	( -0.186, 0.091), 0.194
	61% to 80% (crippled)	Responders, n/N (%)	30/31 (96.77%)	23/28 (82.14%)	1.856	14.63%	(-0.04, 0.335), 0.031*
		Responders with ≥30% improvement, n/N (%)	8/31 (25.81%)	1/31 (3.57%)	2.372	22.24%	(0.019, 0.425), 0.008**

No adverse events were reported in this study. Following treatment initiation, neither was any concomitant/rescue medication required for any indication nor were there any clinically significant changes in hemogram, liver and kidney function tests, or electrolyte levels.

## Discussion

LBP is a self-limiting musculoskeletal condition that poses significant challenges to patients in performing daily activities. Management is, therefore, crucial and emphasis is required to select the appropriate NSAID. In the study population of this trial, the age group mostly affected by LBP was 41-50 years, similar to that observed in earlier studies from India [14]. The results of this study are also in agreement with a recent report wherein LBP burden was shown to peak between 40 and 50 years, as well as data reported in GBD 2017 wherein YLDs were found to peak at 45-49 years of age [15]. Age-standardized prevalence of LBP and YLDs was reported to be higher in females based on GBD 2017 [15]. A female preponderance was also observed in this study as well as in earlier studies on acute LBP [5,14] and in a systematic review from India [4].

This study showed that after 28 days of treatment, the overall percentages of decrease (from baseline) in Wong-Baker Faces Pain score and ODI was significant in both treatment groups and comparable between the groups. However, a higher proportion of subjects with very severe pain or functional disability showed improvement when treated with the DFC compared to the TFC. We have come across only one study that reported the efficacy of the test DFC used in this study. Priyanka et al. [5] in 2022 reported faster relief of recent-onset painful muscle spasms upon treatment with FDC of etoricoxib and thiocolchicoside compared to thiocolchicoside monotherapy in adult patients within 65 years of age. The authors reported adverse events such as gastritis, nausea, itching, and drowsiness in subjects treated with the DFC [5]. On the contrary, no adverse events were encountered in this study. Placebo-controlled trials have previously shown a significant reduction in pain scores upon treatment with etoricoxib [16,17] in patients with chronic pain; the efficacy of etoricoxib was also proven by other studies in chronic LBP [18] and acute LBP [14]. Earlier trials of thiocolchicoside have proven its efficacy/safety with a significant advantage over comparators used [19-22]. This study, together with evidence from the literature, further strengthens the efficacy of FDC of etoricoxib and thiocolchicoside in LBP. Results from this study also proved that the test medication was safe to be used. We did not observe any adverse events, requirement of concomitant/rescue medications, or any clinically significant laboratory test results. As reviewed and discussed in detail by Montazeri and Mousavi, improvement in quality of life has been reported to be the highest in patients with the greatest relief in pain [23]. Taken together, the FDC of etoricoxib (60 mg) and thiocolchicoside (4 mg) is expected to enhance the quality of life in patients. However, further studies are required to establish this firmly.

In this study, we followed subjects for 28 days which is longer than the earlier studies from India that evaluated patients for seven days [5,14]. Further, we enrolled subjects from two study sites while the earlier studies were based on data collected from single centers [5,14]. Both these studies were conducted with ≤100 patients [5,14] whereas the current study was initiated with a total of 200 subjects, 100 in each group. However, all three studies were open-label in design. The limitations of the current study are its small sample size and its open-label design. Further blinded studies conducted across multiple centers with larger populations and longer durations of follow-up are expected to overcome the limitations and strengthen the findings of the current study.

## Conclusions

Based on the results from this study, it can be concluded that the FDC of etoricoxib (60 mg) and thiocolchicoside (4 mg) is effective and safe to be used in adult patients with recent-onset LBP. The overall efficacy of the DFC was found to be comparable to that of the reference TFC used. However, the data support an advantage of the DFC over the TFC in patients with very severe LBP.
